# Factors that distinguish opioid withdrawal during induction with buprenorphine microdosing: a configurational analysis

**DOI:** 10.1186/s13722-022-00336-z

**Published:** 2022-10-04

**Authors:** Adams K. K., Miech E. J., Sobieraj D. M.

**Affiliations:** 1 University of Connecticut School of Pharmacy, 69 N Eagleville Rd Unit 3092, Storrs, CT 06269 USA; 2 Regenstrief Institute, Center for Health Services Research, IN, USA

**Keywords:** Opioid use disorder, Buprenorphine, Configurational comparative methods, Systematic review

## Abstract

**Background:**

Novel buprenorphine dosing strategies have emerged with an aim to transition patients from opioid agonists to buprenorphine without prerequisite opioid withdrawal. We applied a configurational approach to a subset of data from our earlier systematic review to answer the following question: when patients received a buprenorphine initiation strategy aimed to eliminate prerequisite withdrawal, what factors consistently distinguished patients that experienced withdrawal during the initiation process from patients that did not?

**Methods:**

From the 24 cases identified by our systematic review, we included cases that were treated using buprenorphine microdosing strategies (oral or transdermal), cases with opioid use disorder, and cases that fully transitioned to buprenorphine without continuing the full opioid agonist. Configurational analysis was used to identify combinations of patient and regimen level factors that uniquely distinguished cases experiencing withdrawal during induction.

**Result:**

Fourteen cases were included in our analysis, of which 9 experienced opioid withdrawal symptoms. Three factors were involved in explaining both the presence and absence of withdrawal symptoms: history of heroin use, history of methadone use, and duration of overlap between buprenorphine and the full opioid agonist during induction. For the presence of withdrawal symptoms, the addition of a fourth factor “buprenorphine starting dose” resulted in a model with perfect consistency and coverage; for the absence of withdrawal symptoms, the addition of a fourth factor “induction duration” similarly resulted in a model with perfect consistency and 80% coverage.

**Conclusion:**

Application of configurational methods allowed synthesis of case reports identified through a systematic review.

**Supplementary Information:**

The online version contains supplementary material available at 10.1186/s13722-022-00336-z.

## Introduction

Novel buprenorphine dosing strategies have emerged with an aim to transition patients from opioid agonists to buprenorphine without prerequisite opioid withdrawal [1–3]. We previously reported results of a systematic review that aimed to evaluate the safety and efficacy of such strategies [4]. The literature meeting inclusion criteria was limited to case reports thus precluded our ability to statistically analyze outcomes using meta-analysis. Based on qualitative evaluation of these cases, it may be reasonable to expect some patients will successfully transition to sublingual buprenorphine with little to no symptoms of withdrawal while using a novel buprenorphine dosing strategy. However, over half of the cases experienced withdrawal symptoms at some point during the induction process. It is unclear what patient or regimen characteristics distinguish patients that experience withdrawal while using these novel dosing strategies [2–6].

The objective of this study is to apply a configurational approach to a subset of data from our earlier systematic review to answer the following question: when patients received a buprenorphine initiation strategy aimed to eliminate prerequisite withdrawal, what factors consistently distinguished patients that experienced withdrawal during the initiation process from patients that did not?

## Methods

### Systematic review

We completed a systemic review which was previously published; here we explain key methods and results. We conducted a literature search of MEDLINE and CENTRAL from 1996 through April 10, 2020, augmented with searches in Google Scholar and www.clinicaltrials. gov. A study (experimental or observational) was included if it was in patients with substance use disorder or chronic pain that were taking a full mu opioid agonist and transitioning to buprenorphine without preceding withdrawal, and reported withdrawal during initiation as an outcome. Withdrawal was defined as a Clinical Opiate Withdrawal Scale (COWS) score of 5 or more. Fifteen case reports/series, reporting 24 unique cases, met our inclusion criteria; no experimental or observational studies were identified [4].

For this present study, we applied additional criteria to create our analytic data set. We began with the 20 cases that were treated using buprenorphine microdosing strategies (oral or transdermal routes) because it was the most common strategy and sample size allowed further analysis [2, 3, 7–15]. We then excluded 4 cases [10–12] treated for pain only without concomitant opioid use disorder (OUD), since we were interested in analyzing opioid withdrawal as an outcome in patients with OUD. Finally, we excluded 2 cases [13, 14] because they never fully transitioned to buprenorphine and continued use of full opioid agonists. The remaining 14 cases comprised our data set for the configurational analysis (Additional file 1: Table S1).

### Configurational analysis

Configurational comparative methods (CCMs) were applied to the cases reports to identify conditions directly linked to experiencing opioid withdrawal. CCMs are an established group of analytic approaches based upon Boolean algebra and a regularity theory of causation. CCMs identify a set of difference-making combinations of conditions that uniquely distinguish cases with the outcome (e.g., presence of opioid withdrawal) from others without the outcome (i.e. absence of opioid withdrawal) [16, 17]. Furthermore, CCMs allow for conjunctivity and disjunctivity; that is, when multiple conditions must appear together in order for the outcome to be present, and when multiple paths lead to the same outcome. Configurational analysis handles each case as a whole entity which preserves the unique interplay of characteristics rather than deconstructing the case into individual characteristics to analyze independently in relation to a dependent variable [17, 18]. Thus, configurational analysis is particularly suitable for this analysis due to its ability to retain the complex and unique structure of each case as it relates to the outcome. In addition, because it applies Boolean algebra and formal logic instead of linear algebra, configurational analysis can be used with both large and small sample sizes [19, 20]. Finally, multiple meta-analyses and systematic reviews have been conducted using configurational methods in the past, including a 2019 Cochrane Review about interventions for self-management of asthma in school settings [21, 22].

### Outcome and factor selection

For the configurational analysis, our outcome of interest was the presence or absence of any withdrawal symptoms during induction as reported in each case.

Our selection of factors to include in the configurational analysis began with previous literature. Characteristics associated with complicated inductions when traditional buprenorphine dosing is used for induction include absence of previous buprenorphine use [23], a history of methadone use [23], benzodiazepine use [23], and heroin adulterated with fentanyl [24–26]. We included these dichotomous factors (i.e., yes/no) in our configurational analysis to explore their presence in cases experiencing withdrawal during buprenorphine microdosing, with exception of use of heroin adulterated with fentanyl; no cases reported this level of detail, so we used history of heroin use instead (Additional file 1: Table S2).

In addition to the factors above, we included two factors related to the buprenorphine microdosing regimen. First, we collected the buprenorphine starting dose in each case. Buprenorphine doses that are too high may risk precipitated withdrawal [27]. We dichotomized cases into low (< 0.5 mg) or high (≥ 0.5 mg) starting dose because 0.5 mg is the smallest sublingual dose that can be reasonably achieved through dosage form manipulation (i.e. cutting of films) in the United States [28]. To achieve a smaller dose, buprenorphine patches or alternative dosage forms are used. Second, we collected the duration of overlap (in days) with buprenorphine and the full opioid agonist. Unoccupied mu opioid receptors with traditional induction is associated with symptoms of cravings and withdrawal [29] and bears the risk of relapse [3]. The optimal overlap period with a microdosing regimen is unknown. Most patients transition over a period of 4 to 8 days. [1] We selected 80% as a threshold after reviewing the spread of data across the included cases while aiming to minimize fragmentation of the data.

### Data analysis

Configurational analysis was conducted using the Coincidence Analysis (“cna”) package (Version 3.2.0) in R (Version 4.1.0) and RStudio (Version 1.4.1717). First, we performed an exploratory data analysis on our entire dataset to inform factor selection, using the same process that has been described in detail in an earlier study [30]. We applied the msc function within the R package “cna” to identify all one, two- and three-condition configurations instantiated within the dataset that met a pre-specified consistency threshold. We started with a consistency threshold of 100%, and looked for configurations that met all of the following criteria: satisfied the consistency threshold; had “best in class” coverage (i.e., higher coverage scores than any other configuration with the same complexity level); and where the same set of factors—when taking on different values—were involved in explaining both the presence and absence of the outcome. If no configurations appeared at the 100% threshold that satisfied all of these criteria, we iteratively lowered the consistency threshold by 5 points and re-ran the analysis until configurations appeared that met all selection criteria. Using this configurational output, we identified a smaller subset of factors to use in the subsequent modeling phase of the configurational analysis. We used the modeling function in the R package “cna” to develop solutions. Final model selection was based on the following criteria: consistency (number of cases covered by the solution that also had the outcome present divided by the total number of cases covered by the solution) of ≥ 80%; coverage (number of cases covered by the solution that also had the outcome present divided by the total number of cases with the outcome present) of ≥ 80%; having a common set of factors involved in explaining both the presence and absence of the outcome (i.e., these factors were consistently linked with the presence of the outcome when they took on certain values, and consistently linked with the absence of the outcome when they took on other values); and alignment with theory, case knowledge and subject matter expertise.

## Results

Nine of the 14 cases experienced opioid withdrawal symptoms. Using a consistency threshold of 100%, the exploratory data analysis revealed three factors with strong connections to both the presence and absence of the outcome: “HX_HEROIN_USE”, “HX_METHADONE_USE”, and “INDUCTION_OVERLAPPED_80PERCPLUS.” The factor selection process also identified one additional factor that combined with these three factors to explain the presence of withdrawal, and one additional factor that combined with these three factors to explain the absence of withdrawal symptoms (Additional file 1: Table S2).

In the subsequent modeling phase, this set of three factors combined with an additional fourth factor—“HIGH_STARTING_DOSE”—to generate a model for “presence of withdrawal symptoms” with 100% consistency and 100% coverage. There was modest model ambiguity, with two solutions mathematically fitting the data equally well that were identical except for a single condition in a single pathway:

CANDIDATE MODEL A HX_HEROIN_USE=1*HX_METHADONE_USE=1 + HX_HEROIN_USE=0*HX_METHADONE_USE=0 + HIGH_STARTING_DOSE=0*INDUCTION_OVERLAPPED_80PERCPLUS=0 <-> WITHDRAWAL=1

CANDIDATE MODEL B HX_HEROIN_USE=1*HX_METHADONE_USE=1 + HX_HEROIN_USE=0*HIGH_STARTING_DOSE=0 + HIGH_STARTING_DOSE=0*INDUCTION_OVERLAPPED_80PERCPLUS=0 <-> WITHDRAWAL=1

Candidate Model A listed above was selected as the final model based on detailed evaluation of the cases covered by each pathway in the context of clinical and theoretical knowledge of opioid use withdrawal and pharmacologic properties of the drugs involved. A solution visualization for this model is displayed in Additional file 2: Fig. S1 This model had three pathways:

1. A history of heroin use combined with a history of methadone use
2. No history of heroin use combined with no history of methadone use
3. Buprenorphine starting dose that was low (< 0.5 mg) combined with overlap of the full opioid with buprenorphine < 80% of the induction duration.

Any of these three pathways was sufficient for the outcome to appear, as shown in Additional file 2: Fig. S1. Overall, this model explained all 9 cases with any withdrawal symptoms with perfect consistency.

In the solution for “no withdrawal symptoms” the set of three factors combined with a different fourth factor—“INDUCTION_8DAYSPLUS”—to generate a model with 100% consistency and 80% coverage. As before, there was modest model ambiguity, with two solutions that mathematically fit the data equally well that were likewise identical except for a single condition in a single pathway:

CANDIDATE MODEL C HX_HEROIN_USE=0*HX_METHADONE_USE=1 + HX_HEROIN_USE=1* HX_METHADONE_USE=0*INDUCTION_OVERLAPPED_80PERCPLUS=1 <-> WITHDRAWAL=0

CANDIDATE MODEL D HX_HEROIN_USE=0*INDUCTION_8DAYSPLUS=1 + HX_HEROIN_USE=1* HX_METHADONE_USE=0*INDUCTION_OVERLAPPED_80PERCPLUS=1 <-> WITHDRAWAL=0

Candidate Model D listed above was selected as the final model based on detailed evaluation of the cases covered by each pathway in the context of clinical and theoretical knowledge of opioid use withdrawal and pharmacologic properties of the drugs involved. A solution visualization for the final negative model is displayed below in Additional file 2: Fig. S2.

This model had two pathways:

1. No history of heroin use combined with induction of 8 days or more
2. History of heroin use combined with no history of methadone use and overlap of the full opioid with buprenorphine ≥ 80% of the induction duration

Any of these two pathways was sufficient for the outcome to appear, as shown in Additional file 2 Fig. S2. Overall, this model explained 4 of 5 cases with no withdrawal symptoms with perfect consistency.

## Discussion

No single condition alone explained either the presence or absence of opioid withdrawal during buprenorphine induction with microdosing.

Our configurational analysis discovered 3 pathways consistent with the presence of opioid withdrawal during buprenorphine induction with microdosing. The presence of both heroin history and methadone history was one pathway. Heroin adulterated with fentanyl and a history of methadone are known factors that result in complicated inductions [23–26], which compound challenges with transitioning patients from full opioid agonists to buprenorphine. Cases reported a wide range of methadone doses (30–160 mg). The second pathway discovered was an absence of both heroin and methadone history. The absence of both opioids suggests a subset of patients with milder OUD compared to other cohorts. Previous literature has identified that patients without prior buprenorphine use are more likely to experience withdrawal due to unfamiliarity with buprenorphine and withdrawal [21]; a patient experienced with induction may be less likely to report withdrawal symptoms if accustomed to the process. This concept can likely be applied here, as patients with milder OUD may report more withdrawal during induction due to novelty of the experience.

Our final pathway to experiencing withdrawal was characterized by a low buprenorphine starting dose (< 0.5 mg) and induction overlap < 80%. To understand this combination of factors fully, we reviewed the 2 cases explained with this pathway further. In one case [3], the patient continued self-administering intranasal heroin as their opioid taper. This was the only case included in our analysis in which this was reported. It would be reasonable to think the clinicians in the case encouraged this patient to taper the illicit heroin as quickly as they were able since encouraging patients to continue illicit drugs in an unsupervised outpatient setting is inconsistent with general practice. Additionally, strength of illicit heroin cannot be determined with certainty and thus the actual taper in this case may not have necessarily reflected the desired or appropriate taper for the true strength of heroin used. In the second case [15], 220 morphine milligram equivalents were tapered over 3 days during a 4-day induction period. This opioid taper is rapid, especially considering 50% of cases in our analysis completed induction over 8 days or longer. The rate of opioid taper over a short period of time likely lead to withdrawal symptoms. As illustrated by these two cases, a low starting dose of buprenorphine is not protective against withdrawal symptoms during an opioid taper that is too rapid.

Solution pathways for the absence of opioid withdrawal during buprenorphine induction with microdosing differed systematically depending on whether a history of heroin use was present or absent. The first pathway was when a history of heroin use was absent together with an induction that lasted 8 days or longer. Heroin adulterated with fentanyl has been shown to cause complicated withdrawals likely due to its lipophilicity, high-potency, large volume of distribution, and protracted clearance; Those that do not use heroin are less likely to experience this outcome of complicated withdrawal [1, 17, 19, 31]. An induction time of 8 days or greater suggests a slower titration. Due to the slow displacement of opioids from the mu receptor, this would be less likely to cause withdrawal symptoms. The second pathway to absence of withdrawal was when a history of heroin use was present, a history of methadone use was absent, and the overlap of buprenorphine with the full opioid was  ≥ 80% of the induction phase. This solution suggests that the subset of patients with history of heroin could still transition to buprenorphine without withdrawal so long as the buprenorphine and full opioid agonist overlap is sufficient to allow for a slow displacement of opioids from the mu receptor; this only occurs in cases when history of methadone use is not present, likely due to methadone’s long half-life and unique pharmacokinetics that make it challenging to transition patients to buprenorphine [32].

Systematic reviews aim to synthesize evidence by “bringing together data from a set of included studies with the aim of drawing conclusions about a body of evidence [33]”. Meta-analysis is often used to synthesize included studies and generate a single effect estimate that quantifies the magnitude and direction of effect of an intervention; for example, in the groups being compared. There are scenarios that preclude meta-analysis, including heterogeneity in the evidence base or situations like ours where only case reports exist, which are uncontrolled without comparator groups. Case reports have an important place in the medical literature and can provide valuable insight into novel treatment strategies for a known condition that may be further explored and generate hypotheses for future experimental studies [34]. In the absence of experimental studies, we believe the case reports identified are an important contribution to the current management of patients with OUD. Configurational methods have been suggested as another approach to synthesizing evidence from a systematic review and are particularly suited in our analysis as a case-oriented method of synthesis [35]. Application of configurational analysis allowed us to understand which combination of factors consistently identified cases with and without withdrawal symptoms when initiating buprenorphine microdosing strategies for OUD. These results can be used to generate hypotheses for future experimental studies to further elucidate microdosing efficacy and safety.

### Practice implications and future research needs

Non-medical literature such as blogs and online discussion threads contain anecdotal patient reports of the benefits of “buprenorphine microdosing” and its utility in preventing opioid withdrawal [36, 37]. Patients who rely on these platforms may develop unrealistic expectations of non-traditional dosing strategies. For the provider who chooses to pursue an alternative buprenorphine induction strategy, having a conversation regarding withdrawal with the patient is critical as some patients will experience withdrawal with these strategies. Unexpected withdrawal, even if less than with traditional induction, may result in frayed patient-provider relationships.

No data exists to explain which patients may experience withdrawal and which patients will not experience withdrawal using buprenorphine microdosing. Future research is needed to evaluate the efficacy and safety of microdosing strategies but to also help define patients that are optimal for this induction strategy. The buprenorphine microdosing method can be used as long as the basic principles are followed [1]. Until then, our analysis provides initial understanding of what pathways distinguish published cases with opioid withdrawal compared to published cases without withdrawal and is an update to existing evidence [1, 4, 25, 38–40]. This insight can better inform provider discussions and patient understanding of expectations during the induction process. Patients who align with our “positive solution” pathways may be expected to report a level of withdrawal with buprenorphine microdosing strategies, while patients who align with our “negative solution” pathways may be less likely to report symptoms of withdrawal.

## Limitations

Our analysis is limited by the information reported within each published case. We cannot rule out publication bias of cases where patients experienced negative outcomes, limiting the evaluation of factors consistent with having withdrawal during these novel buprenorphine inductions. If information to discern a factor within a case was not explicitly stated, we took it as a “no”. In some cases, we relied on descriptive statements in the report to determine presence of withdrawal symptoms although it is unknown if these statements were founded on validated tools or were the opinion of the patient or provider. We initially aimed to evaluate presence of at least moderate withdrawal symptoms according to the COWS, but only two cases described enough detail to discern this. Factors such as benzodiazepine use [23], historical buprenorphine use [23], patient deviation from the induction protocol, and moderate withdrawal were either not reported or were present at low numbers that precluded analysis. Finally, buccal dosing strategies have emerged since the systematic review upon which this analysis was based and thus were not considered in the analysis [41].

## Conclusion

During buprenorphine induction with microdosing strategies in patients with opioid use disorder, three factors appeared in models for both the presence and absence of withdrawal symptoms: history of heroin use, history of methadone use, and duration of overlap between buprenorphine and the full opioid agonist during induction. Cases that experienced withdrawal and those that did not were identified with perfect consistency when a fourth factor was combined, buprenorphine starting dose and induction duration, respectively. Application of configurational methods allowed synthesis of case reports identified through a systematic review.

## Supplementary Information

**Additional file 1: Table S1.** Cases included in the analysis. **Table S2.** Factors and definitions.

**Additional file 2: Figure S1.** Solution visualization for positive model (presence of any withdrawal symptoms); model consistency = 100% and model coverage = 100%. **Figure S2.** Solution visualization for negative model (absence of any withdrawal symptoms); model consistency = 100% and model coverage = 80%.

## Data Availability

Data is available from the authors upon request.

## Abbreviations

CCM: Configurational comparative method
COWS: Clinical opiate withdrawal score
OUD: Opioid use disorder
