# Supplementary material for: Factors that distinguish opioid withdrawal during induction with buprenorphine microdosing: a configurational analysis
Source: Addict Sci Clin Pract. 2022 Oct 4;17:55. doi: 10.1186/s13722-022-00336-z (PMC9531395; doi:10.1186/s13722-022-00336-z)
Supplement: Supplementary file 1 — Additional file 1: Table S1. Cases included in the analysis. Table S2. Factors and definitions. [file 13722_2022_336_MOESM1_ESM.pdf]

Table 1. Cases included in the analysis

| Author, Case # | History of opioid use | Opioid regimen prior to induction | Initial buprenorphine dose, (mg) | Overlap time | Time to complete induction |
|---|---|---|---|---|---|
| Hämmig, R, Case 1 | Heroin | Heroin | 0.2 | 5d | 9d |
| Hämmig, R, Case 2 | Diacetylmorphine, methadone, heroin | Methadone moderate dose + Diacetylmorphine | 0.2 | 28d | 29d |
| Jafari, S Case 1 | Methadone, illicit opioids | Methadone high dose | 0.5 | 4m | 4m |
| Klaire, S Case 1 | Heroin | Heroin + short acting opioids | 0.25 | 4d | 5d |
| Klaire, S Case 2 | Heroin | Short acting opioids | 0.5 | 2d | 3d |
| Martin, L Case 1 | Methadone and buprenorphine | Short acting opioids | 0.5 | 12d | 14d |
| Raheemullah, A Case 1 | Heroin | 220mg morphine equivalents* | 20 ug/hr | 3d | 4d |
| Saal, D Case 2 | Short acting opioids; buprenorphine use | Short acting opioids | 10 ug/hr | 5d | 6d |
| Saal, D Case 3 | Heroin; methadone | Kratom | 10 ug/hr | 4d | 5d |
| Saal, D Case 4 | Long acting opioids + short acting opioids | Long acting opioids + short acting opioids | 10 ug/hr | 3d | 7d |
| Sandhu, R Case 1 | Short acting opioids, heroin | Short acting opioids | 0.25 | 6d | 7d |
| Terasaki, D Case 1 | Heroin, buprenorphine | Methadone low dose | 0.5 | 7d | 8d |
| Terasaki, D Case 2 | Heroin, methadone | Methadone moderate dose + short acting opioids | 0.5 | 7d | 11d |
| Terasaki, D Case 3 | Heroin | Methadone low dose + short acting opioids | 0.5 | 7d | 8d |

Abbreviations: d=day; m=month; mg=milligram; ug/hr = microgram per hour
*specific regimen was not reported

Table 2. Factors and definitions

| Factor Name | Definition and Coding |
|---|---|
| WITHDRAWAL | 0 = no withdrawal reported<br>1= withdrawal reported |
| HX_HEROIN_USE | 0 = no history of heroin use<br>1 = history of heroin use |
| HX_METHADONE_USE | 0 = no history of methadone use<br>1 = history of methadone use |
| HIGH_STARTING_DOSE | 0 = buprenorphine starting dose <0.5mg<br>1 = buprenorphine starting dose ≥0.5mg |
| INDUCTION_OVERLAPPED_80PERCPLUS | 0 = overlap between buprenorphine and full opioid agonist <80% of induction period<br>1 = overlap between buprenorphine and full opioid agonist ≥80% of induction period |
| INDUCTION_8DAYSPLUS | 0 = induction period <8 days<br>1 = induction period ≥8 days |
