# Supplementary material for: Factors that distinguish opioid withdrawal during induction with buprenorphine microdosing: a configurational analysis
Source: Addict Sci Clin Pract. 2022 Oct 4;17:55. doi: 10.1186/s13722-022-00336-z (PMC9531395; doi:10.1186/s13722-022-00336-z)
Supplement: Supplementary file 2 — Additional file 2: Figure S1. Solution visualization for positive model (presence of any withdrawal symptoms); model consistency = 100% and model coverage = 100%. Figure S2. Solution visualization for negative model (absence of any withdrawal symptoms); model consistency = 100% and model coverage = 80%. [file 13722_2022_336_MOESM2_ESM.pdf]

Figure 1. Solution visualization for positive model (presence of any withdrawal symptoms); model consistency = 100% and model coverage = 100%.

Case_ID	WITHDRAWAL	Hx_HEROIN_USE	Hx_METHADONE_USE	HIGH_STARTING_DOSE	INDUCTION_OVERLAPPED_80PERCPLUS
Hämmig, R, Case 2	1	1	1	0	1
Terasaki_1	1	1	1	1	1
Terasaki_2	1	1	1	1	0
Terasaki_3	1	1	1	1	1
Saal_3	1	1	1	0	1
Hämmig_1	1	1	0	0	0
Raheemullah_1	1	1	0	0	0
Saal_4	1	0	0	0	0
Saal_2	1	0	0	0	1
Jafari_1	0	0	1	1	1
Klaire_1	0	1	0	0	1
Klaire, S Case 2	0	1	0	1	0
Martin_1	0	0	1	1	1
Sandhu_1	0	1	0	0	1

Figure 2. Solution visualization for negative model (absence of any withdrawal symptoms); model consistency = 100% and model coverage = 80%.

Case_ID	WITHDRAWAL	INDUCTION_8DAYSPLUS	Hx_HEROIN_USE	Hx_METHADONE_USE	INDUCTION_OVERLAPPED_80PERCPLUS
Jafari_1	0	1	0	1	1
Martin_1	0	1	0	1	1
Klaire_1	0	0	1	0	1
Sandhu_1	0	0	1	0	1
Klaire, S Case 2	0	0	1	0	0
Hämmig_1	1	1	1	0	0
Hämmig, R, Case 2	1	1	1	1	1
Terasaki_1	1	1	1	1	1
Terasaki_2	1	1	1	1	0
Terasaki_3	1	1	1	1	1
Raheemullah_1	1	0	1	0	0
Saal_2	1	0	0	0	1
Saal_3	1	0	1	1	1
Saal_4	1	0	0	0	0
